# Aligning research to meet policy objectives for migrant families: an example from Canada

**DOI:** 10.1186/1478-4505-7-15

**Published:** 2009-06-10

**Authors:** AJ Gagnon, MP Joly, J Bocking

**Affiliations:** 1 School of Nursing & Department of Obstetrics and Gynecology, McGill University, 3506 University Street, Montreal, Canada; 2 McGill University Health Centre, 687 Pine Avenue West F2.27, Montreal, Canada; 3 Department of Sociology, McGill University, Montreal, 855 Sherbrooke Street West, Canada

## Abstract

**Background:**

'Evidence-based policy making' for immigrants is a complicated undertaking. In striving toward this goal, federal Canadian partners created the *Metropolis Project *in 1995 to optimize a two-way transfer of knowledge (researchers – policy makers) within five Canadian Centres of Excellence focused on migrants newly arrived in Canada. Most recently, *Metropolis *federal partners, including the Public Health Agency of Canada, defined one of six research priority areas as, immigrant 'families, children, and youth'. In order to build on previous work in the partnership, we sought to determine what has been studied within this research-policy partnership about immigrant 'families, children, and youth' since its inception.

**Methods:**

Annual reports and working papers produced in the five Centres of Excellence between 1996–2006 were culled. Data on academic works were extracted, results coded according to eleven stated federal policy priority themes, and analyzed descriptively.

**Results:**

139 academic works were reviewed. All federal priority themes, but few specific policy questions were addressed. The greatest volume of policy relevant works were identified for *Services *(n = 42) and *Education and Cultural Identity *(n = 39) priority themes.

**Conclusion:**

Research conducted within the last 10 years is available to inform certain, not all, federal policy questions. Greater specificity in federal priorities can be expected to more clearly direct future research within this policy-research partnership.

## Background

Evidence-based policy-making has been cited as important in several countries and for a variety of different issues [1-5]. Several models of research utilization/translation have been described, including 'knowledge-driven', 'problem-solving', 'interactive', 'political', 'tactical', and 'enlightenment' models[6]. Successful projects with regards to dissemination have been found to be those that address a topical issue, are precisely defined, are of national significance, have the commitment of those carrying it out, have university involvement, substantial funding, a team structure, and experienced investigators working in a supportive environment[5]. Case studies suggest that research translation is enhanced if policy-makers are included in the development of the research and if researchers ensure their research is included in policy discussions[5].

To optimize a two-way transfer of knowledge (researchers – policy makers), the Social Sciences and Humanities Research Council (SSHRC), Citizenship and Immigration Canada (CIC) and a consortium of federal agencies and departments in Canada created the *Metropolis Project * in 1995 with funding provided by SSHRC and the consortium. The federal consortium is led by CIC and currently includes: Atlantic Canada Opportunities Agency, Canada Border Services Agency , Canada Economic Development for the Regions of Quebec , Canada Mortgage and Housing Corporation, , CIC , Federal Economic Development Initiative for Northern Ontario , Human Resources and Social Development Canada,  Department of Justice Canada , Public Health Agency of Canada,  Public Safety Canada,  Canadian Mounted Police , The Rural Secretariat of Agriculture and Agri-Food Canada , SSHRC , and Statistics Canada .

*Metropolis *serves as a network of researchers, policy-makers, and migrant advocates at both international (21 countries) and national levels. Canadian *Metropolis *encompasses five University-Based Centres of Excellence (Atlantic, Quebec, Ontario, Prairie, British Columbia) comprised of local networks of researchers, policy-makers from different levels of government (i.e. federal, provincial, and/or municipal), and practitioners/non-governmental organizations that focus on immigration and diversity in Canadian cities. They are managed by Centre Management Committees and Governance Boards, led by Centre Directors, and are divided into research theme areas (i.e. 'domains'), which are led by Domain Coordinators. This work is guided by the policy-research priorities identified by the federal funders through interactions with stakeholders and partners[7].

All research undertaken under the aegis of the Canadian National *Metropolis Project *occurs through the Centres of Excellence and their domains. The vast majority of specific research projects are funded through peer-review mechanisms at the Centre-level, with fewer through a national competition peer-reviewed for science through SSHRC mechanisms as well as an ad hoc committee created to assess policy relevance of the subset found by SSHRC to be scientifically sound.

*Metropolis *has been funded in three phases, with Project assessments undertaken at the mid- and end-points of each phase (and planned for the current Phase 3)[8,9]. Continued funding for each phase and subsequent phase was based on evidence of success of conducting policy-relevant research within each Centre. This was unable to be measured directly and was therefore based on: the number of researchers and community organizations involved, the number of projects underway, the number of employment and training opportunities offered to graduate students, and stated positive views of the Project in questionnaire responses and focus group meetings with federal funding partners.

In these assessment exercises, *Metropolis *was felt to contribute significant, new and useful knowledge on the subjects of immigration and integration. *Metropolis *research projects, developed in collaboration with community and policy partners, were felt to provide relevant information and tools to develop and assess policies and improve services. Assessments also found that the Centres are providing multidisciplinary training focused on community policy development, that they are intensively involved in disseminating research results to target audiences, and that they are providing expert advice in public debates on immigration issues. Assessments have noted the impressive scope and diversity of community involvement with the Centres.

In a more recent assessment using detailed interviews about the *Metropolis Project *with fourteen Deputy Ministers or equivalent senior policy makers and nine leaders of think tanks and research institutes working on immigration and diversity, *Metropolis *was generally considered by senior policy makers and think tank leaders as "an undoubted success in its decade of development and activity in stimulating immigration/diversity research and its transfer to policy making, with a high international reputation", although some critics stated being "unaware or unconvinced of specific identifiable policy results"[10].

Areas recently defined as priority by federal partners include: (1) citizenship and social, cultural and civic integration; (2) economic and labour market integration; (3) family, children and youth; (4) housing and neighbourhoods; (5) justice, policing, and security; and (6) welcoming communities in attracting, integrating, and retaining newcomers and minorities. Six national Priority Leaders, drawn from the Centres, ensure that their priority areas are more visible across *Metropolis *events and activities. These Leaders are also tasked with working with the *Metropolis *Secretariat and federal partners to transfer findings to policy-makers more regularly and effectively than in previous phases.

Health was purposely not labelled as a separate priority because as a signatory to the Ottawa Charter for Health Promotion (1986)[11], Canada sees health defined broadly. The PHAC therefore saw health as affecting and being affected by each of the six priorities identified by the federal partners.

Senior Canadian policy makers see immigration and diversity as of critical and growing importance for Canada's future[10]. Migration to Canada occurs in large numbers[12], with migrant children and youth (0–24 years old) constituting a high proportion of the total; 36.5% – almost 92,000 – of all new permanent residents in 2006[12]. These figures highlight the importance of the family, children, and youth policy priority identified in the *Metropolis Project*. In addition, this priority area offers the opportunity to examine more closely a wide range of policy themes in which an interplay between those policies and health are likely. The policy priority themes and questions defined within the family, children, and youth priority area are detailed in Table 1 [see additional file 1]. Themes include: migration decisions, cultural identity, education and cultural identity, educational outcomes, civil participation and work, extracurricular activities, migration, gender, health services, and intergenerational dynamics. The existence of this policy priority area, with its specific themes, and policy questions, offered us a unique opportunity to empirically examine what has been studied within this research-policy partnership about immigrant 'families, children, and youth' since its inception. This paper seeks to highlight what was learned.

## Methods

### Study outcomes

Any outcomes of works related to families, children and youth were of interest.

### Type of exposure and study population

Being a newcomer was the exposure of interest. Therefore, any academic work of families, children, or youth who had migrated to Canada from any other country was included.

### Type of study designs

We included all research design types.

### Search strategy

Identifying academic works for inclusion

Annual reports produced in the context of the five centres of excellence between 1996–2006 were culled.

### Appropriateness of works assembled

Duplicate works and those that were clearly off-topic were eliminated. A list was created for works identified in annual reports but which could not be located for review.

### Data coding

Data were extracted on year of academic work, language of work, journal of publication, type of academic work (abstract, journal article, unpublished review, major research paper, thesis, national *Metropolis *conference, presentation, report, research capsules, working paper, and 'unknown'), number of newcomers under study and geographic coverage of database (i.e. international, national, regional, or local).

The study populations were categorized by age based on Statistics Canada's population breakdown . This includes: <1 year, 1–4, 5–9, 10–14, 15–19, 20–24, 25–64, 65+. If a study's sample population covered more than one category, any category with any amount of representation was included and a note was made as to what portion of the age was involved (e.g. a study with a sample population of 18 years and up was coded under 15–19, 20–24, 25–64 and 65+).

Several migration labels may have been named in the same work thus we decided to code only those used in reporting results. These were classified into general categories (based on frequency of occurrence) defined as follows: (1) country of birth/foreign-born: any label which required data on country of birth to define it; (2) ethnicity: a commonly undefined term used by authors, which could include ethnicity, ethnic group, ethnic mix, race; (3) nationality: a commonly undefined term used by authors, which could include national origin, citizen, citizenship; (4) language: any label which required data on language to define it; (5) refugee: the term as used by authors may include those who left home unwillingly and/or having been to resettlement camps; and (6) immigrant status: the term as categorized by the authors and may include labels such as "first generation", "second generation", "third generation", "fourth generation", "visible minority", "family class", "landed", "economic", or "undocumented" 'immigrant'.

The Memorandum of Understanding (MOU) between the Social Sciences and Humanities Research Council and Citizenship and Immigration Canada for research to be conducted within the *Metropolis *Project on Family, Children, and Youth from 2007–2012[7] was used to define eleven federal priority themes. They include: (1) migration decisions, (2) cultural identity, (3) education and cultural identity, (4) educational outcomes, (5) civil participation and work, (6) extracurricular activities, (7) mental health, (8) health and movement, (9) health and gender, (10) services, and (11) intergenerational dynamics. Specific priority questions within each group (total n = 58) were given a code. Priority themes and specific questions can be found in Table 1 [see additional file 1].

Results from each academic work were categorized under the appropriate priority theme(s). If works addressed issues unrelated to a specific policy question but related to the priority theme, they were categorized as 'other' within the theme. A single study could have had results that were categorized under more than one theme. If no priority themes were addressed the academic work was classified as "other".

Outcome results for each work were subsequently coded as 'worse', 'better', 'mixed' or 'not different', as reported by authors for newcomer compared to Canadian-born. For results that did not compare newcomer to Canadian-born, outcomes were coded as descriptive comparative (if comparing amongst newcomer populations) or descriptive. The coding 'absent' indicated academic works that did not report results (e.g. research in process, certain abstracts). 'Worse' and 'better' categories could have included 'not different' on certain outcomes but those that were different, were either all worse or all better, respectively. Only the 'mixed' category included one or more outcomes benefiting newcomers and one or more benefiting non-newcomers in the same work (and may have included outcomes that were not different). These results were coded within the federal priority categories listed above.

### Data management

Data abstracted from each work were entered into an Excel database; categorization of all outcomes into priority themes and questions was repeated for verification. Multiple works presenting data from the same study were combined into a single row of data in order to avoid over-weighting any particular study within the overall results.

### Assessment of heterogeneity

We assumed the studies included in this review to be heterogeneous. Our inclusion and exclusion criteria were broad to include works of newcomers from a range of source countries worldwide. We classified data into categories of exposure, outcome, and data sources to be as inclusive as possible. The extent of heterogeneity was assessed by examining the methods, outcomes and populations included in the works.

### Study sample

139 works met our inclusion criteria; 119 others could not be located (87% were "project" descriptions).

### Analyses

Works were described with respect to the variables defined previously. Each of the outcome categories were classified by the number of works reporting results as being worse, better, not different, mixed, absent, descriptive or descriptive comparative.

## Results

Table 2 [see additional file 2] describes the characteristics of included works. The majority of works meeting our inclusion criteria were produced in English (74%), and were working papers (36%). The review incorporated data from 29,639 newcomers. 66% of the studies reporting sample sizes, reported having <100 newcomers and nearly 34% reported having >100 newcomers. About 69% of the study population was younger than 25 (0–14 yrs – 26%, 15–24 yrs – 43%), while 31% focused on adult and senior populations (25–64 yrs – 24%, 65 yrs and above- 7%). Local geographic areas were the most common sources of data (50%). Works reported comparisons using a variety of migration labels, although 'immigrant status' was the most common (33%), followed by 'country of birth' (23%). Of all works reviewed, 37 (27%) were published journal articles.

**Figure 1:**
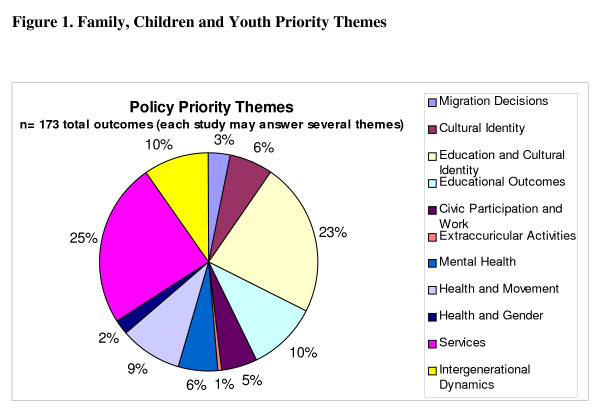
**Family, Youth, and Children Priority Themes**. This pie-chart details the breakdown of priority themes

Figure 1 shows the percentage of results of all works reviewed that were categorized under each priority theme. Services (n = 42) and Education and Cultural Identity (n = 39) were most common, however all priority themes had at least one result.

Tables 3 and 4 [see additional file 3 and additional file 4] present summaries of the results for newcomer populations in qualitative and quantitative works, respectively. These show that a range of outcomes was reported, with several comparisons often presented within the same work. 110 (64.7%) of the reported outcomes do not address specific federal priority questions but rather, the general theme of the priority, while 17 did not address any federal priority questions. The number of outcomes categorized under individual questions within a priority theme (including 'other' categories) ranged from 0 to 30. Qualitative works were available to inform the eleven priority themes.

Quantitative works may offer an estimate of the prevalence of an issue or the relative importance of it in newcomer versus non-newcomer groups and hence offer a perspective on equity [11]. The studies included in this review were found to be heterogeneous on several factors and thus require caution in interpreting their results. With this caveat in mind, overall assessments of reported quantitative results were available for 5 priority themes – educational outcomes, civic participation and work, mental health, health and movement, and services (see Table 4 [see additional file 4]).

## Discussion

### Principal findings

All federal priority themes but few specific policy questions were addressed in the works produced within the *Metropolis *partnership between 1996 and 2006 on family, children, and youth new to Canada. The greatest volume of policy-relevant academic works were identified for 'Services and Education' and 'Cultural Identity'. Most studies were descriptive and non-comparative in nature, with small study populations drawn from local areas.

### Strengths and weaknesses

We sought to gather information on existing evidence available to inform recently defined federal priorities for newcomer family, children, and youth in Canada within a research-policy partnership. We directly assessed the link between work produced over a 10-year period and specifically defined federal policy themes and questions. A broad search for academic works produced within the partnership was undertaken using annual reports, centre web sites, and direct contact. No exclusion criteria were applied and any relevant work was included in this review.

This review was not exempt of possible bias, especially given the large number of academic works that could not be located, although the majority of these appear to have been ongoing projects.

### Relationship to other studies

Reports have described models for research utilization[6] and suggested approaches for translating research into policy[5]. Empirical data comparing research conducted directly against policy priorities for research were not identified in the literature. The fact that research over a 10-year period was able to provide some evidence to inform newly defined policies may be partially due to the fact that two-way communication between researchers and policy makers was in place as a cornerstone of the project. In policy-makers' view of their use of evidence, this two-way personal communication was most commonly cited as a suggestion to improve the use of research evidence[4].

### Implications and future research

This review of 10 years' of academic output to inform recently defined federal policies concerning aspects of newcomer family, children, and youth suggests that although some research evidence is already available, several priority questions are unanswered. In discussions of these results with policy-makers, they felt that the specific priority question was less important than the priority theme, that the questions serve to exemplify what might be examined under each theme (especially given the fluid nature of policy priorities at any one point in time), and that as long as the priority theme is being examined, responses to specific questions would be useful but are not required. For researchers who may want to use these questions to guide their research programs, the questions may be too specific and there may be too many of them (n = 58); using them as examples may serve researchers best. More frequent direct contact between researchers and policy-makers (already in play under Metropolis Phase 3) can be expected to facilitate a greater depth of understanding by researchers of the kinds of questions that can inform policy; and by policy-makers of inherent time and other constraints in conducting research. Together, researchers and policy-makers can more narrowly define specific priority policy questions within the general priority area of migrant family, children, and youth, which will, over the intermediate-term, offer the policy-maker a greater volume of research in selected areas rather than a smaller number of projects in many areas which makes drawing conclusions much more difficult.

This review clearly shows the heavy use of qualitative designs in academic work in this field. Researchers applying this methodology will need to determine how best to transmit the knowledge gained by this approach to policy-makers since policy-makers are likely more familiar with the use of representative population-based quantitative data (e.g. census data) to inform their policies. In contrast, research related to migration (perhaps because of its link to ethnicity) has often been addressed within disciplines that commonly use qualitative methodologies such as in-depth interviews and ethnographies to answer their research questions. The goals of these design types have often been to try to understand why or how from that individual's or community's perspective rather than what or how much. Policy-makers will need to be clear on which type of answer they are seeking and qualitative researchers will need to explain to policy-makers how this type of information can help policy-makers create better policy.

## Conclusion

Research conducted within a research-policy partnership over the last 10 years is already available to inform certain, not all, Canadian federal policy questions related to Family, Children, and Youth. Further specification of federal priority questions can be expected to more clearly direct future research in this area.

## Competing interests

The authors declare that they have no competing interests.

## Authors' contributions

AG led the conceptualization of the project and analysis and interpretation of data, as well as the drafting and the critical revision of the manuscript. MPJ performed statistical analyses and interpreted the data, and drafted and edited parts of the manuscript. JB was involved in the conception of the database, the study design and the editing of the manuscript. All authors read and approved the final manuscript.

## Authors' informations

Anita Gagnon is the Priority Leader for Family, Children, and Youth for the Metropolis Project

## Supplementary Material

Additional file 1 **Table 1: Detailed Policy Priority Questions and their Priority Themes**. This table outlines specific priority questions and themes. Click here for file

Additional file 2 **Table 2. Characteristics of Academic Works of Families, Children, and Youth Newcomers to Canada**. Characteristics of other studies regarding families, children, and youth newcomers to Canada. Click here for file

Additional file 3 **Table 3. Family, Children and Youth Priorities Examined in Qualitative Works**. Characteristics of qualitative studies on family, children, and youth priorities. Click here for file

Additional file 4 **Table 4. Family, Children and Youth Priorities Examined in Quantitative Works**. Characteristics of quantitative studies on family, children, and youth priorities. Click here for file
