# Supplementary material for: Aligning research to meet policy objectives for migrant families: an example from Canada
Source: Health Res Policy Syst. 2009 Jun 10;7:15. doi: 10.1186/1478-4505-7-15 (PMC2711941; doi:10.1186/1478-4505-7-15)
Supplement: Additional file 1 — Table 1: Detailed Policy Priority Questions and their Priority Themes. This table outlines specific priority questions and themes. [file 1478-4505-7-15-S1.doc]

**Table 1. Detailed Policy Priority Questions and their Priority Themes**

| **Migration decisions (MD)** | |
| --- | --- |
| **MD1** | What factors influence the migration decisions of individuals and families? |
| **MD2** | Do these factors change depending on one’s life course? |
| **MD3** | What role do children and youth play in the decision-making process? |
| **MD4** | What are the means though which migrants assemble information upon which to make their migration decisions? |
| **MD5** | How is done with respect to questions surrounding the recognition of foreign credentials, family reunification, refugee claims and naturalization, among others? |
| **MD6** | Does the government of Canada through its policies and programs (e.g. Canada Orientation Abroad, Immigration Portal, have the ability to significantly alter the migration decisions of migrants? |
| **Cultural identity (CI)** | |
| **CI1** | What is the sense of identity, attachment and belonging to Canada, as well as to their heritage, among immigrant and refugee children and youth |
| **CI2** | How are these multiple attachments and belongings reflected in their identities? |
| **CI3** | Do foreign-born and Canadian-born minority children and youth negotiate these complex identities in the same fashion? |
| **CI4** | If not, what explains the differences? |
| **CI5** | What indicators can be used to ascertain levels of identity, attachment and belonging, whether these levels change over time, and what factors account for this change? |
| **CI6** | What views do children and youth have on both their culture of origin and their society in respect to identity? |
| **CI7** | How do children and youth construct an identity for themselves, a sense of home and belonging, and a sense of origin and descent in both contexts? |
| **CI8** | How and what kind of relationship is maintained with the place of origin of children and youth during the migration process, and does such a relationship influence the social interactions among children, between children and their social environment? |
| **CI9** | Are there gender-specific differences concerning the construction of identity by children and youth in the course of migration |
| **Education and cultural identity (ECI)** | |
| **ECI1** | How does the education system’s accommodation of religious practices and/or celebrations affect feelings of belonging among young people? |
| **ECI2** | What is the role of educational policies and school practices in the formation of multiple and trans-nationalist citizenships among immigrant children and youth, and in their formation of a sense of belonging to Canada? |
| **ECI3** | What is the role of schools in both official language acquisition, and heritage language maintenance? |
| **ECI4** | What is the role of both school-based and non-school-based heritage language programs (i.e. church- and-community-based language for immigrant children and youth development of multilingual/multicultural programs for children belongings and identities? |
| **ECI5** | To what extent and how do children experience xenophobia, and how do they cope with hostility and lack of acceptance? |
| **ECI6** | How do they create social niches where they can feel at home? |
| **ECI7** | What is the role of language acquisition in seniors’ integration? |
| **Other** | Language policy; multilingualism; language official/heritage; language barrier, language learning |
| **Educational outcomes (EO)** | |
| **EO1** | Are there differential outcomes for immigrant, refugee and minority children and youth? |
| **EO2** | What accounts for differential educational aspirations, rates of early school leaving and/ or higher levels of educational attainment for some? |
| **EO3** | Are there differences in outcomes for first, 1.5, and second generation children and youth? |
| **EO4** | Are there inter-group differences in immigrant, refugee and minority children and how are these addressed in schools? |
| **EO5** | How does the interplay between the particular cultural background of their society of origin and the integration strategies and practices in a particular cultural background of their society of origin and the integration strategies and practices in a particular host society affect the integration and/or educational achievement of children and youth? |
| **Civil participation and work (CPW)** | |
| **CPW1** | Does participation in school and/or civil society vary among the Canadian-born, immigrant, refugee and minority children and youth? |
| **CPW2** | Are there differences in school readiness, in rates of voluntarism, or in rates of civic participation? |
| **CPW3** | Are there inter-group differences in immigrant, refugee and minority children and youth in school and /or civic society participation and what accounts for these differences? |
| **CPW4** | What is the role of citizenship education in nurturing civic and public engagement in immigrant children youth? |
| **CPW5** | What other models of learning and job training (i.e. Apprenticeship are available for them and how can such models be made available in communities, and neighborhoods where they live? |
| **Extracurricular activities (EA)** | |
| **EA1** | What is the role of both school-based and non-school based creative opportunities in enhancing and nurturing creativity in immigrant and minority children and youth? |
| **EA2** | What non-school based learning opportunities, recreational/sports and creative/artistic programs are available for immigrant, refugee, and minority children and youth? |
| **EA3** | Do they influence their integration into larger communities and into the larger society? |
| **EA4** | What lifelong learning opportunities are provided to immigrant seniors? |
| **Mental Health (MH )** | |
| **MH1** | What are the key determinants of the mental and emotional well-being of newcomer, second generation, and/or minority children and youth in Canada |
| **MH2** | Are there mental health problems related to the social isolation of seniors? |
| **MH3** | If so, what factors can help mitigate this risk? |
| **Health and movement (HM)** | |
| **HM1** | Are their mental and emotional stresses that coincide with migration to Canada, and id so, does this vary by immigration category (e.g. family class, refugee, and/or by length of residence in Canada? |
| **HM2** | What pre-migratory conditions and experiences influence post-migratory health across the life course? |
| **0ther** | Physical and emotional health related to motherhood and parenting; social development; reported health status, health behaviors; traditional cures, types of medication and culture; immigration health policy with regards to women’s reproductive health |
| **Health and Gender (HG)** | |
| **HG1** | Are there differential mental and physical health effects across the life course on women and men of immigration to Canada? |
| **HG2** | If differences are evident. What are the factors that lead to these disparities? |
| **Services (S)** | |
| **S1** | How effective are public health interventions and prevention messages at reaching immigrant children and youth? |
| **S2** | How does the efficacy of programs for marginalized youth in Canada differ for immigrant youth? |
| **S3** | What are the implications of the different cultural and religious backgrounds and values of immigrants to Canada for public health services and programs such as immunization, healthy sexuality, etc? |
| **S4** | What services are available for immigrant and minority children and youth and what is the impact of these services on these groups? |
| **S5** | What role do families – both extended and nuclear – play in facilitating integration? |
| **S6** | Do immigrants who are accepted through family reunification appear to face fewer, greater or different integration challenges than immigrants and refugees who enter through other streams? |
| **S7** | What are the service gaps and how should these gaps be addressed? |
| **S8** | Are immigrant and minority children effectively integrated into daycare services? |
| **0ther** | Health care needs and health care responsiveness; health care utilization; culture, language and health care providers; auxiliary work |
| **Intergenerational dynamics (ID)** | |
| **ID1** | How do family dynamics change in the process of integration to the host society? |
| **ID2** | What are the principal intergenerational conflicts faced by immigrant and minority youth? |
| **ID3** | How does their immigration and integration experience differ from that of their parents? |
| **ID4** | How do interactions and tensions between peer group environments and home socialization affect children? |
| **ID5** | What policy and program interventions might most effectively mitigate these impacts? |
| **ID6** | What is the role that “language gap” both in official and heritage languages plays in the change of family dynamics? |
| **ID7** | Do seniors experience more integration than other newcomers and/or minorities? |
| **0ther** | Motherhood; mother identities; mothering and schooling; generational values; parenting; family and gender expectations; generational household and caregiving; family and social support |
