# Supplementary material for: Aligning research to meet policy objectives for migrant families: an example from Canada
Source: Health Res Policy Syst. 2009 Jun 10;7:15. doi: 10.1186/1478-4505-7-15 (PMC2711941; doi:10.1186/1478-4505-7-15)
Supplement: Additional file 2 — Table 2. Characteristics of Academic Works of Families, Children, and Youth Newcomers to Canada. Characteristics of other studies regarding families, children, and youth newcomers to Canada. [file 1478-4505-7-15-S2.doc]

**Table 2. Characteristics of Academic Works of Families, Children, and Youth Newcomers to Canada**

| **Characteristic of work** | **Reviewed (n=139)** | |
| --- | --- | --- |
|  | N | % |
| Language: |  |  |
| English | 103 | 74% |
| French | 36 | 26% |
| English and French | 139 | 100% |
|  |  |  |
| Types of academic work: |  |  |
| Abstract | 6 | 4% |
| Journal article | 37 | 27% |
| Unpublished review | 2 | 1% |
| Major research paper | 2 | 1% |
| Thesis | 2 | 1% |
| National Metropolis Conference | 1 | 1% |
| Presentation | 1 | 1% |
| Report | 24 | 17% |
| Research capsule | 2 | 1% |
| Working Paper | 50 | 37% |
| Unknown | 12 | 9% |
|  |  |  |
| N of newcomers in work: | n=42[[1]](#footnote-2) |  |
| 0-99 | 28 | 66% |
| 100-499 | 4 | 10% |
| 500-999 | 2 | 5% |
| 1000+ | 8 | 19% |
|  |  |  |
| Age groupings (yrs): | n=119[[2]](#footnote-3) |  |
| <1 | 2 | 2% |
| 1-4 | 2 | 2% |
| 5-9 | 7 | 6% |
| 10-14 | 19 | 16% |
| 15-19 | 29 | 24% |
| 20-24 | 23 | 19% |
| 25-64 | 29 | 24% |
| 65+ | 8 | 7% |
|  |  |  |
| Geographic Coverage: | n=124[[3]](#footnote-4) |  |
| Local | 61 | 50% |
| Regional | 24 | 19% |
| National | 30 | 24% |
| International | 9 | 7% |
|  |  |  |
| Migration Labels: | n=128[[4]](#footnote-5) |  |
| Country of birth/foreign-born | 30 | 23% |
| Ethnicity | 15 | 12% |
| Nationality | 0 | 0% |
| Foreigner | 1 | 1% |
| Language | 20 | 16% |
| Refugee | 19 | 15% |
| Immigrant | 43 | 33% |

1. Only 42 academic works have included the number of migrants studied. [↑](#footnote-ref-2)
2. The age groupings have been reported 119 times, as one study may have used more than one age grouping. [↑](#footnote-ref-3)
3. Not every academic works have mentioned the geographical coverage of their study. Abstracts for instance may not necessarily give this information. [↑](#footnote-ref-4)
4. Among the academic works, the migration labels have been used 128 times. Again, one study might have used more than one label. [↑](#footnote-ref-5)
