# Supplementary material for: Aligning research to meet policy objectives for migrant families: an example from Canada
Source: Health Res Policy Syst. 2009 Jun 10;7:15. doi: 10.1186/1478-4505-7-15 (PMC2711941; doi:10.1186/1478-4505-7-15)
Supplement: Additional file 3 — Table 3. Family, Children and Youth Priorities Examined in Qualitative Works. Characteristics of qualitative studies on family, children, and youth priorities. [file 1478-4505-7-15-S3.doc]

# **Table 3. Family, Children and Youth Priorities Examined in Qualitative Works**^a^

| **Priority themes and specific questions addressed** ^b^ | **Results**  **N**^c^ | **Number of Descriptive**  **Studies** | **Number of**  **Descriptive-comparative**  **Studies** |
| --- | --- | --- | --- |
| Migration Decisions |  |  |  |
| MD1 | 4 | 4 | 0 |
| MD3 | 1 | 1 | 0 |
| Other | 1 | 1 | 0 |
|  |  |  |  |
| Cultural Identity |  |  |  |
| CI1 | 4 | 4 | 0 |
| CI2 | 1 | 1 | 0 |
| CI6 | 2 | 2 | 0 |
| CI7 | 1 | 1 | 0 |
| Other | 3 | 3 | 0 |
|  |  |  |  |
| Education and Cultural Identity |  |  |  |
| ECI3 | 1 | 1 | 0 |
| ECI4 | 1 | 1 | 0 |
| ECI5 | 3 | 2 | 1 |
| Other | 29 | 27 | 2 |
|  |  |  |  |
| Educational Outcomes |  |  |  |
| EO1 | 1 | 1 | 0 |
| EO2 | 2 | 1 | 1 |
| Other | 6 | 6 | 0 |
|  |  |  |  |
| Civic Participation and Work |  |  |  |
| CPW4 | 1 | 1 | 0 |
| Other | 5 | 5 | 0 |
|  |  |  |  |
| Extracurricular Activities |  |  |  |
| Other | 1 | 0 | 1 |
|  |  |  |  |
| Mental Health |  |  |  |
| MH1 | 3 | 3 | 0 |
| Other | 3 | 2 | 1 |
|  |  |  |  |
| Health and Movement |  |  |  |
| HM2 | 1 | 1 | 0 |
| Other | 9 | 9 | 0 |
|  |  |  |  |
| Health and Gender |  |  |  |
| HG1 | 2 | 2 | 0 |
| Other | 2 | 2 | 0 |
|  |  |  |  |
| Services |  |  |  |
| S7 | 6 | 6 | 0 |
| S8 | 2 | 2 | 0 |
| Other | 25 | 22 | 3 |
|  |  |  |  |
| Intergenerational Dynamics |  |  |  |
| ID1 | 2 | 2 | 0 |
| ID2 | 1 | 1 | 0 |
| ID3 | 1 | 1 | 0 |
| Other | 13 | 13 | 0 |
|  |  |  |  |
| Other | 13 | 12 | 1 |

^a^ Specific references to inform this table (n=110) can be obtained from the authors.

^b^ See Table 1 for specific priority questions under each theme; works with no results presented were not included in these tables, but were reviewed

^c^ A single study could include more than one comparison
