# Supplementary material for: Aligning research to meet policy objectives for migrant families: an example from Canada
Source: Health Res Policy Syst. 2009 Jun 10;7:15. doi: 10.1186/1478-4505-7-15 (PMC2711941; doi:10.1186/1478-4505-7-15)
Supplement: Additional file 4 — Table 4. Family, Children and Youth Priorities Examined in Quantitative Works. Characteristics of quantitative studies on family, children, and youth priorities. [file 1478-4505-7-15-S4.doc]

# **Table 4. Family, Children and Youth Priorities Examined in Quantitative Works^a,b^**

| **Priority themes and specific questions addressed** ^c^ | **Results**  **n**^d^ | **Number of “Worse”** | **Number of “Better”** | **Number of “Mixed”** | **Number of “No difference”** |
| --- | --- | --- | --- | --- | --- |
| Educational Outcomes  EO1 | 3 | 0 | 1 | 2 | 0 |
| EO2 | 2 | 2 | 0 | 0 | 0 |
| Other | 3 | 1 | 0 | 2 | 0 |
|  |  |  |  |  |  |
| Civic Participation and Work |  |  |  |  |  |
| Other | 1 | 1 | 0 | 0 | 0 |
|  |  |  |  |  |  |
| Mental Health |  |  |  |  |  |
| MH1 | 1 | 0 | 0 | 1 | 0 |
| Other | 1 | 0 | 0 | 0 | 1 |
|  |  |  |  |  |  |
| Health and Movement |  |  |  |  |  |
| Other | 3 | 2 | 1 | 0 | 0 |
|  |  |  |  |  |  |
| Services |  |  |  |  |  |
| Other | 4 | 0 | 3 | 0 | 1 |
|  |  |  |  |  |  |
| Other | 1 | 1 | 0 | 0 | 0 |

^a^ Specific references to inform this table (n=18) can be obtained from the authors.

^b^ Results as reported by authors for newcomer compared to Canadian-born; ‘worse’ and ‘better’ categories could have included ‘not different’ on certain outcomes but those that were different, were either all worse or all better, respectively; the ‘mixed’ category included one or more outcomes benefiting newcomers and one or more benefiting non-newcomers in the same work (and may have included outcomes that were not different), ‘descriptive’ if no comparison group, ‘descriptive comparative’ if comparison between newcomer groups.

^c^ See Table 1 for specific priority questions under each theme; works with no results presented were not included in these tables, but were reviewed

^d^ A single study could include more than one comparison
